# The Cellular Prion Protein Interacts with the Tissue Non-Specific Alkaline Phosphatase in Membrane Microdomains of Bioaminergic Neuronal Cells

**DOI:** 10.1371/journal.pone.0006497

**Published:** 2009-08-04

**Authors:** Myriam Ermonval, Anne Baudry, Florence Baychelier, Elodie Pradines, Mathéa Pietri, Kimimitsu Oda, Benoît Schneider, Sophie Mouillet-Richard, Jean-Marie Launay, Odile Kellermann

**Affiliations:** 1 Différenciation cellulaire et Prions, Institut Pasteur, Département de Biologie Cellulaire et Infection, Paris, France; 2 CNRS FRE 2937, Villejuif, France; 3 FRE 2942, Oncologie virale, Villejuif, France; 4 Division of Oral Biochemistry, Niigata University Graduate School of Medical and Dental Sciences, Niigata City, Japan; 5 AP-HP Service de Biochimie, U942 INSERM Hôpital Lariboisière, Paris, France; 6 Pharma Research Department, F. Hoffmann-La-Roche, Basel, Switzerland; Instituto Oswaldo Cruz and FIOCRUZ, Brazil

## Abstract

**Background:**

The cellular prion protein, PrP^C^, is GPI anchored and abundant in lipid rafts. The absolute requirement of PrP^C^ in neurodegeneration associated to prion diseases is well established. However, the function of this ubiquitous protein is still puzzling. Our previous work using the 1C11 neuronal model, provided evidence that PrP^C^ acts as a cell surface receptor. Besides a ubiquitous signaling function of PrP^C^, we have described a neuronal specificity pointing to a role of PrP^C^ in neuronal homeostasis. 1C11 cells, upon appropriate induction, engage into neuronal differentiation programs, giving rise either to serotonergic (1C11^5-HT^) or noradrenergic (1C11^NE^) derivatives.

**Methodology/Principal Findings:**

The neuronal specificity of PrP^C^ signaling prompted us to search for PrP^C^ partners in 1C11-derived bioaminergic neuronal cells. We show here by immunoprecipitation an association of PrP^C^ with an 80 kDa protein identified by mass spectrometry as the tissue non-specific alkaline phosphatase (TNAP). This interaction occurs in lipid rafts and is restricted to 1C11-derived neuronal progenies. Our data indicate that TNAP is implemented during the differentiation programs of 1C11^5-HT^ and 1C11^NE^ cells and is active at their cell surface. Noteworthy, TNAP may contribute to the regulation of serotonin or catecholamine synthesis in 1C11^5-HT^ and 1C11^NE^ bioaminergic cells by controlling pyridoxal phosphate levels. Finally, TNAP activity is shown to modulate the phosphorylation status of laminin and thereby its interaction with PrP.

**Conclusion/Significance:**

The identification of a novel PrP^C^ partner in lipid rafts of neuronal cells favors the idea of a role of PrP in multiple functions. Because PrP^C^ and laminin functionally interact to support neuronal differentiation and memory consolidation, our findings introduce TNAP as a functional protagonist in the PrP^C^-laminin interplay. The partnership between TNAP and PrP^C^ in neuronal cells may provide new clues as to the neurospecificity of PrP^C^ function.

## Introduction

The cellular prion protein PrP^C^ is a ubiquitous glycoprotein anchored at the plasma membrane through a glycosylphosphatidylinositol (GPI) lipid moiety. It is abundantly expressed in neurons of the central nervous system (CNS), which are the main target of transmissible spongiform encephalopathies (TSE). The conversion of PrP^C^ into an abnormal conformer, PrP^Sc^, prone to aggregation, is a hallmark of prion diseases. In addition to having a genetic or sporadic origin like other neurodegenerative disorders, prion diseases have the unique peculiarity to be transmissible, the PrP^Sc^ conformer being the main if not the only component of the pathogenic agent [1].

The absolute requirement of PrP^C^ for the development of prion diseases is well established. However, the precise role of this protein is yet to be fully determined. Its identification should help to understand how the pathogenic isoforms interfere with the cellular function of normal PrP^C^ [2]. Recent data have shown that PrP^C^ plays a role in cell signaling and cell adhesion and may act as a membrane receptor or co-receptor [3]–[5], consistent with its extra-cellular orientation. Interestingly, PrP^C^ is expressed at the plasma membrane in sub-domains enriched in cholesterol and sphingolipid [6] described as rafts and known to play a role in cellular events such as sorting of membrane constituents and signal transduction [7]. While the location of PrP^C^ in lipid rafts is suspected to be required for its conversion into PrP^Sc^ [8], [9], it could also have implications as to PrP^C^ function.

Attempts to identify physiological ligands or partners that could bring light on PrP^C^ function have relied on different approaches (two hybrid techniques, immunoprecipitation of cellular PrP^C^ complexes, complementary hydropathy analyses…). Only some of the interactions have been confirmed and/or shown to have functional relevance at a cellular level [10]. PrP^C^ associates with molecular chaperones such as BiP, grp94, protein disulfide isomerase or calnexin, required for the proper folding of glycoproteins [11]. Another PrP^C^-interacting molecule is the stress inducible protein I (STI-I) chaperone, described as having a neuroprotective action [12]. PrP^C^ partners also include proteins involved in signal transduction such as synapsin 1, important for synapse formation and neurotransmitter release, the adaptor Grb2 molecule [13] and the protein casein kinase 2, CK2 [14]. Also, adhesion molecules such as laminin and the 37/67 kDa laminin receptor have been shown to interact with PrP^C^ [15]–[17], with heparan sulphated molecules acting as intermediates [18]. Graner et al. have notably reported on the impact of the PrP^C^-laminin interaction on neurite outgrowth [16]. Chemical cross-linking analyses have identified the neuronal adhesion molecule, NCAM, as another PrP^C^ interacting protein [19]. This interaction appears to sustain the recruitment of NCAM into lipid rafts, the activation of the Fyn tyrosine kinase and N-CAM-mediated neurite outgrowth [20]. The latter observation recalls our demonstration that antibody mediated PrP^C^ cross-linking triggers Fyn activation in 1C11-derived neuronal cells via the lipid raft protein caveolin [3].

In order to search for PrP^C^ partners, we took advantage of the 1C11 neuronal differentiation model [21], which previously allowed us to substantiate a role for PrP^C^ in signal transduction. Upon appropriate induction, the 1C11 neuroepithelial cell line engages into a neuronal differentiation program. Nearly 100% cells acquire the overall functions of serotonergic (1C11^5-HT^) or noradrenergic (1C11^NE^) neurons, within 4 or 12 days, respectively. By unraveling some signal transduction events instructed by PrP^C^, our previous work has pointed to the implication of the cellular prion protein in cell homeostasis [3], [22]–[24]. In 1C11^5-HT^ and 1C11^NE^ differentiated cells, the implementation of a PrP^C^-caveolin-Fyn platform on neuritic extensions controls multiple pathways converging to the MAP kinases, ERK1/2. Furthermore, in addition to its proper signaling activity, PrP^C^ modulates the agonist-induced response of the three serotonin receptors coupled to G-proteins present on 1C11^5-HT^ cells, themselves regulating the overall serotonergic functions [25]. Interestingly, this modulatory role of PrP^C^ is also restricted to fully differentiated cells and is caveolin-dependent.

The neuronal specificity of PrP^C^ signaling function may rely on some of the numerous isoforms and/or glycoforms of this protein resulting from proteolytic cleavage and heterogenous glycosylation [26], [27]. It could also depend on PrP^C^ partners induced during the bioaminergic programs and/or recruited into lipid rafts. The purpose of the present study was to search for PrP^C^ partners in lipid microdomains of differentiated neuronal cells. By an approach combining immunoprecipitation of PrP^C^ from lipid rafts and mass spectrometry analysis, we identify the tissue non-specific alkaline phosphatase (TNAP) as interacting with PrP^C^ in membrane microdomains of both 1C11^5-HT^ and 1C11^NE^ cells. TNAP is a GPI membrane-bound alkaline phosphatase (AP) expressed as three distinct isoforms found respectively in liver, kidney and at a high level in bone where it plays an essential role in osteogenesis [28]. Recent data identified TNAP in different cell types of the brain [29], [30]. While its role is still elusive, it has been proposed to participate to neurotransmission [29].

Here, we show that TNAP is induced along either the serotonergic or noradrenergic differentiation program of 1C11 cells. This ectoenzyme is active under physiological conditions and may participate in bioamine synthesis. Besides, we provide evidence that the PrP^C^-interacting protein laminin is a substrate for TNAP in 1C11-derived neuronal cells, and that, by modulating the phosphorylation level of laminin, TNAP impacts on the interaction between PrP and laminin.

## Results

### PrP^C^ partitions in lipid rafts irrespective of the differentiation state of 1C11 cells

**Figure 1:**
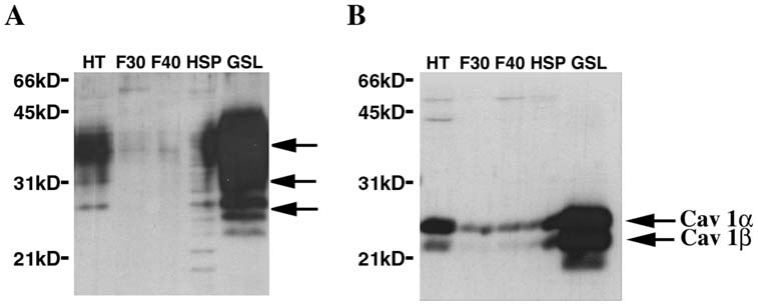
PrP^C^ partitions in lipid rafts of 1C11 cells. Proteins (10 µg) from different fractions of 1C11 cells isolated on a discontinuous sucrose gradient, i.e. total homogenate (HT), the 30% (F30) and 40% (F40) soluble layers, insoluble pellet (HSP) and the raft (GSL) fraction, were separated on a 12% SDS-PAGE and analyzed by western blotting. The presence of PrP^C^ (A) and caveolin 1 (B) was assessed using SAF32 and C060 monoclonal antibodies, respectively. Arrows indicate the different forms of PrP^C^ (non-, mono and biglycosylated) and the α and β chains of caveolin 1.

The presence of PrP^C^ in lipid rafts of 1C11 precursor, 1C11^5-HT^ or 1C11^NE^ fully differentiated cells, was assessed using TritonX-100 insoluble glycosphingolipid (GSL) rich microdomains isolated by flotation on sucrose gradient and solubilized in 6% SDS. As revealed by Western blot, PrP^C^ majorly segregated into the GSL fraction of 1C11 cells (Fig. 1A) and its neuronal progenies (not shown). A similar result was obtained for caveolin 1, a marker of caveolae which are subtypes of lipid rafts (Fig. 1B).

**Figure 2:**
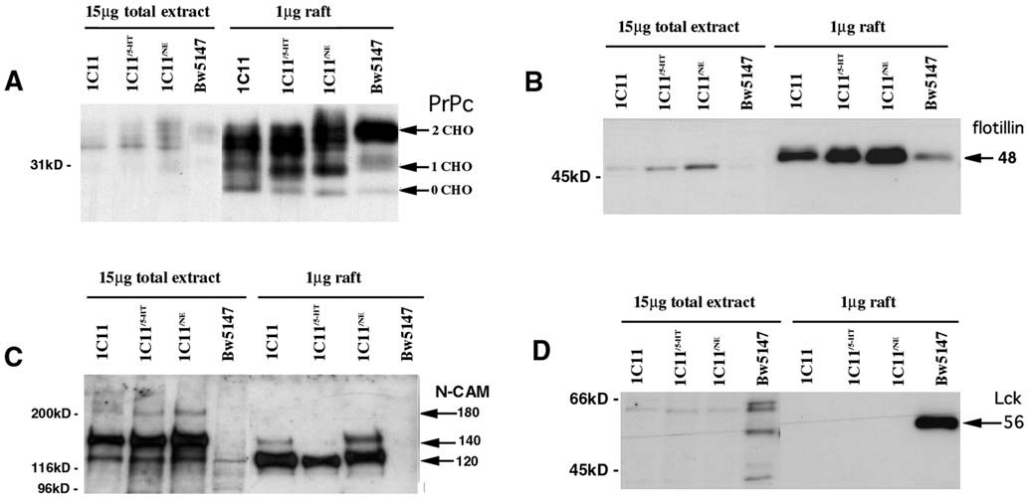
PrP^C^ is enriched in lipid rafts irrespective of the differentiated state of 1C11 cells. Proteins of total extracts (15 µg) and lipid rafts (1 µg) from 1C11 cells, their neuronal 1C11^5-HT^ and 1C11^NE^ derivatives, and Bw5147 lymphoid cells (used as control) were resolved by 12% SDS-PAGE and analyzed by Western blot. Detection of PrP^C^ (A) and other raft markers, i.e. flotillin (B), the NCAM isoforms (C) and Lck kinase (D). A 100- to 200-fold increase in the amount of PrP^C^, flotillin and the GPI-anchored 120 kDa isoform of NCAM was observed in GSL fractions in 1C11 cells whatever their differentiation state. As expected, Lck kinase, was only present in lipid rafts of lymphoid cells. Of note, the B, C and D panels were obtained by stripping and reprobing the same blotted nitrocellulose membrane.

To evaluate the degree of PrP^C^ enrichment in lipid rafts, comparative analyses were performed by Western blot using proteins of raft preparations (1 µg) and total extracts (15 µg) from 1C11 precursor, 1C11^5-HT^ (day 4) and 1C11^NE^ (day 12) cells. Irrespective of the differentiation state, a 100- to 200-fold increase in the amount of PrP^C^ was observed in GSL fractions (Fig. 2A). A similar enrichment was observed for other proteins specific of lipid rafts such as flotillin (Fig. 2B), caveolin 1 (Fig. 1B and not shown) and the GPI-anchored 120 kDa isoform of NCAM (Fig. 2C), the latter two described as interacting with PrP^C^ [3], [19]. While NCAM120 was enriched in lipid rafts, it is noteworthy that the 140 kDa transmembrane form of NCAM was predominant in total extracts.

These data indicate that the enrichment of PrP^C^ in lipid rafts is independent from the differentiation state, precursor vs neuronal, of 1C11 cells.

### PrP^C^ interacts in microdomains of 1C11^5-HT^ and 1C11^NE^ neuronal cells with an 80 kDa protein identified as the tissue non-specific alkaline phosphatase, TNAP

**Figure 3:**
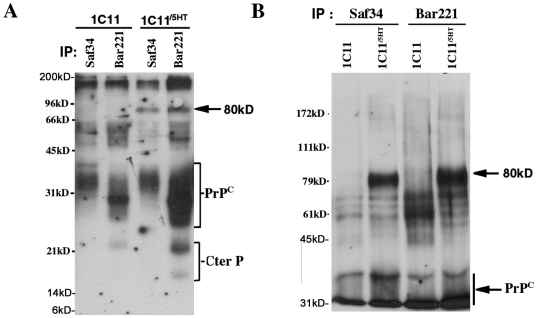
PrP^C^ interacts with a 80 kDa protein in microdomains of 1C11^5-HT^ and 1C11^NE^ neuronal cells. Lipid rafts from 1C11^5-HT^, 1C11^NE^ and 1C11 precursor cells were prepared after biotinylation of membrane proteins. PrP^C^ was immunoprecipitated either with an anti-N-ter monoclonal antibody (SAF34) or an anti-C-ter antibody (Bar221) covalently linked to sepharose beads. The biotinylated PrP^C^ complexes were separated on a 12% SDS-PAGE to visualize all the PrP^C^ species (A) and on an 8% gel to better separate PrP^C^ co-immunoprecipitated protein of higher apparent molecular mass (B). Biotinylated proteins were revealed with streptavidin peroxidase conjugate. The 80 kDa protein that co-immunoprecipitates with PrP^C^ in 1C11^5-HT^ is indicated by an arrow.

In order to seach for potential PrP^C^ partners in such specialized microdomains, plasma membrane proteins of 1C11, 1C11^5-HT^ and 1C11^NE^ cells were labelled with biotin before raft preparation. GSL fractions were dissolved in non ionic detergent (1% TritonX-100) to maintain some protein interactions and heated for 1 hour at 37°C to allow extraction of proteins from membrane cholesterol. Antibodies recognizing either N-ter (SAF34) or C-ter (Bar221) epitopes of PrP^C^ were covalently linked to sepharose beads and used to immunoprecipitate PrP^C^. The immunoprecipitated complexes were resolved on a 12% SDS-PAGE (Fig. 3A). The biotinylated full-length mono or bi-glycosylated PrP^C^ species were immunoprecipitated with both antibodies in 1C11^5-HT^ differentiated cells as well as in 1C11 precursor cells. The glycoforms corresponding to the N-terminally truncated fragments of PrP^C^ were recovered with the Bar221 antibody only. A few other biotinylated proteins appeared to be co-precipitating with PrP^C^ both in 1C11 precursor cells and in bioaminergic neuronal cells. These include proteins with an apparent molecular mass between 45–65 kDa (fig. 3A and B) as well as proteins of high molecular weight (around 200 kDa). Interestingly, using either anti-N-ter or anti-C-ter PrP^C^ antibodies, an 80 kDa biotinylated protein was co-precipitated with PrP^C^ in lipid rafts of 1C11^5-HT^ and 1C11^NE^ cells. The presence of this 80 kDa protein within PrP^C^ complexes appears to depend on neuronal differentiation, since we failed to detect this protein co-precipitating with PrP^C^ in lipid rafts of the 1C11 neuroepithelial precursor (Fig. 3 and data not shown).

**Figure 4:**
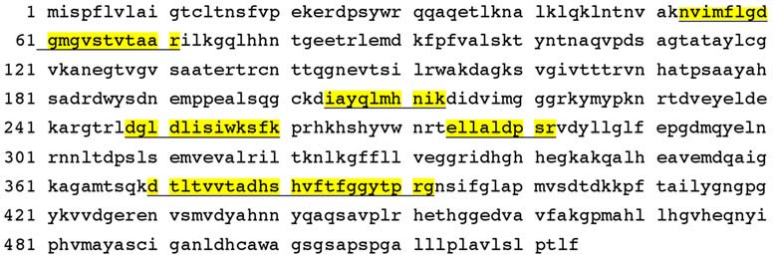
The 80 kDa PrP^C^ partner is identified as TNAP by mass spectrometry. The position of the 5 peptides identified by mass spectrometry (LC/MS/MS) with the best scores, is highlighted along the mouse TNAP protein sequence.

Mass spectrometry analysis was then carried out to define the identity of this 80 kDa PrP^C^ partner. Lipid rafts were prepared from 1C11^5-HT^ and 1C11^NE^ cells as well as from 1C11 precursor. PrP^C^ complexes were immunoprecipitated as above and separated on an 8% SDS-PAGE allowing a better resolution in the 50–100 kDa range of proteins as exemplified in Figure 3B. Proteins of 80 kDa apparent molecular mass were trypsin-digested and analyzed with a LC/MS/MS instrument. The experimental peptide fragments were confronted to the NCBI non-redundant mouse database. Five peptides (aa_53–71_, aa_204–213_, aa_248–260_, aa_274–282_, aa_370–392_) that matched different regions of the TNAP sequence (Fig. 4) were identified with a high score (60.17) in 1C11^5-HT^ and 1C11^NE^ cells. In contrast, TNAP peptides were not detected in immunoprecipitates from 1C11 precursor cells.

We took advantage of an anti-TNAP antibody [31] to further study the TNAP-PrP^C^ interaction. Performing the reverse immunoprecipitation with the anti-TNAP antibody did not allow a clear detection of associated proteins (data not shown). The anti-TNAP polyclonal antibody may promote a destabilisation of TNAP-PrP^C^ complexes. It is also worth noting that under conditions where biotinylated TNAP was easily revealed in PrP^C^ immune-complexes by streptavidin, the anti-TNAP antibody failed to yield a signal at 80 kDa. This suggests that sensitive technics (biotinylation, mass spectrometry analysis) are required to reveal TNAP co-precipitating with PrP^C^.

**Figure 5:**
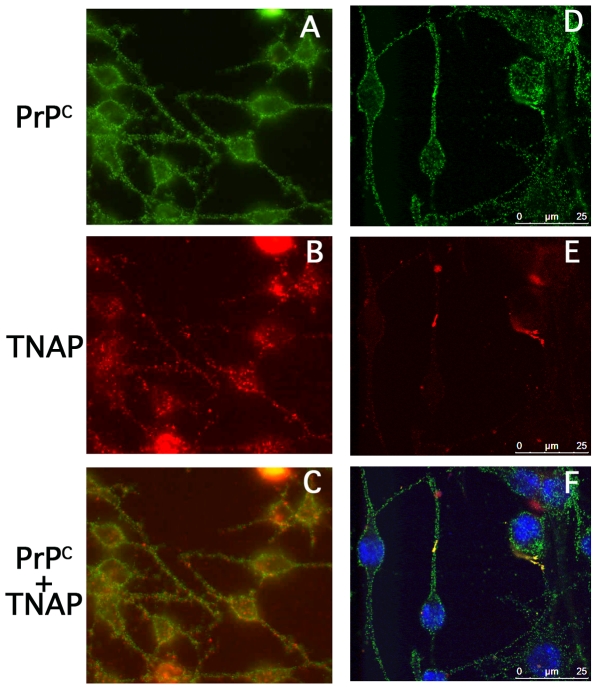
Co-localization of PrP^C^ and TNAP at the surface of 1C11^5-HT^ cells. Membrane immunofluorescence was performed on 1C11^5-HT^ live cells cultured on glass coverslips. PrP^C^ (A and D) was stained with the monoclonal SAF32 antibody and TNAP (B and E) with polyclonal anti-TNAP antibodies and revealed with alexa green or red labeled secondary antibodies, respectively. A superimposition of the two fluorescence labelings is shown in panels (C) and (F). Panels (A), (B) and (C) were obtained by epifluorescence and (D), (E) and (F) were obtained by sequential acquisition on a scanning confocal microscope.

We next evaluated the distribution of TNAP at the cell surface of 1C11^5-HT^ serotonergic cells by immunofluorescence. TNAP antibodies yielded a punctate staining of the membrane, both on cell bodies and on neurites (Fig. 5, B and E). Such a labeling was reminiscent of the PrP^C^ staining (Fig. 5, A and D). The superimposition of the two stainings showed a partial co-localization of TNAP and PrP^C^ at the surface of 1C11^5-HT^ neuronal cells (Fig. 5C) that is confirmed by scanning confocal analysis (fig. 5F).

As a whole, these data introduce TNAP as a neurospecific PrP^C^ partner, in lipid rafts of either 1C11 serotonergic or noradrenergic progenies. They also indicate that membrane-bound TNAP and PrP^C^ are located in close vicinity within raft domains.

### TNAP expression is restricted to 1C11^5-HT^ serotonergic and 1C11^NE^ neuronal cells

**Figure 6:**
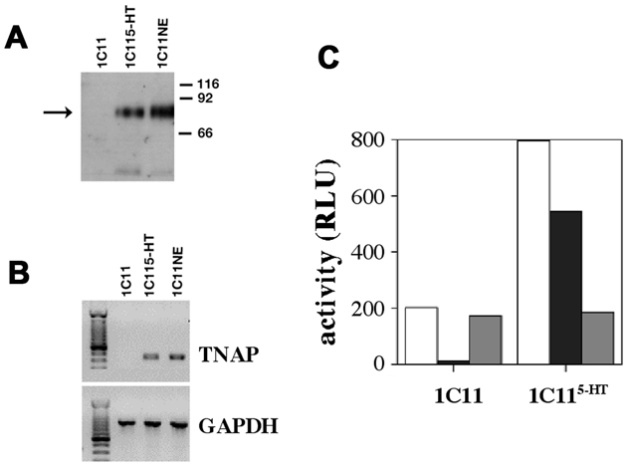
The expression of a functional TNAP is restricted to differentiated serotonergic and noradrenergic 1C11 derived-cells. In (A), the presence of TNAP in 1 µg of lipid rafts prepared from 1C11 induced or not to differentiate was revealed by western blot analysis using an anti-TNAP specific antibody. In (B), the expression of TNAP mRNAs was evaluated by PCR analysis. TNAP (upper panel) or GAPDH (lower panel) specific fragments were obtained after amplification by PCR of cDNA synthesized from mRNA isolated from the 1C11 precursor and the differentiated 1C11^5-HT^ and 1C11^NE^ cells. In (C), phosphatase activity at the surface of 1C11 and 1C11^5-HT^ cells was measured by luminescence using the CSPD substrate and expressed as relative luminescent unit (RLU). White bars correspond to total phosphatase activities, black bars to the activity measured in the presence of 1 mM orthovanadate and grey bars in the presence of 5 mM tetramisol.

The restriction of TNAP interaction with PrP^C^ to neuronal 1C11^5-HT^ and 1C11^NE^ cells prompted us to investigate whether TNAP was present in microdomains of 1C11 precursor cells. Direct LC/MS/MS analysis of trypsin-digested 80 kDa raft components did not allow the identification of any peptides corresponding to mouse TNAP sequence in 1C11 precursor cells, while it firmly confirmed the presence of TNAP in microdomains of serotonergic and noradrenergic differentiated cells. Indeed, three (aa_53–71_, aa_370–391_, aa_392–407_) and five (aa_53–71_, aa_247–257_, aa_273–282_, aa_370–391_, aa_392–407_) TNAP derived-peptides were respectively identified in microdomains of serotonergic (score of 40.2) and noradrenergic (score of 70.3) neuronal cells (see Fig. 4 for residues numbering). In addition, Western blot analysis using a TNAP specific antibody [31] also revealed a unique band of 80 kDa apparent molecular mass in the rafts of 1C11^5-HT^ and 1C11^NE^ cells, which was absent in 1C11 precursor cells (Fig. 6A).

The absence of the TNAP protein in 1C11 cells correlated with a lack of TNAP gene expression as assessed by RT-PCR analysis. As shown in Figure 6B, TNAP transcripts were below detectable levels in the 1C11 precursor and were abundant in 1C11^5-HT^ and 1C11^NE^ neuronal cells.

As a whole, these results show that 1C11 precursor cells lack TNAP and that the expression of this ectoenzyme is restricted to 1C11^5-HT^ and 1C11^NE^ neuronal cells.

### A functional TNAP is induced during the differentiation of 1C11^5-HT^ and 1C11^NE^ cells

**Figure 7:**
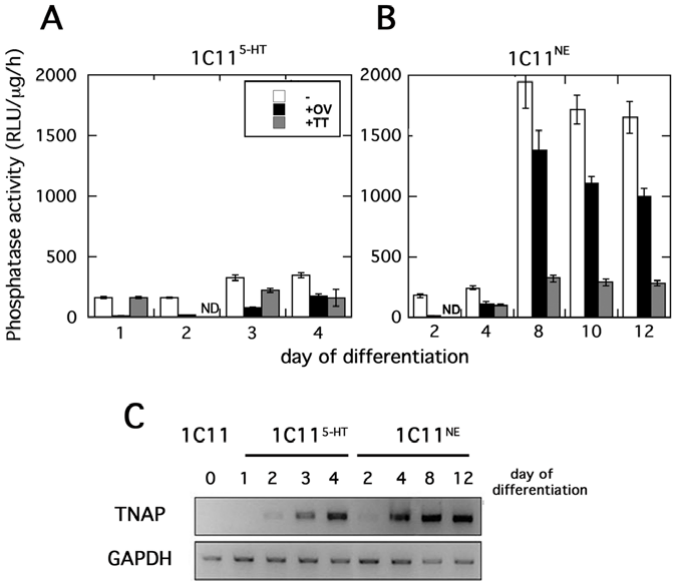
TNAP activity is implemented during serotonergic and noradrenergic differentiation of 1C11 cells. TNAP activity was evaluated in 1C11^5-HT^ (A) and 1C11^NE^ (B) cells during the kinetics of differentiation. The histograms represent the level of phosphatase activity (RLU/µg prot/h) monitored without (white bars) or with orthovanadate (black bars) or tetramisol (grey bars) phosphatase inhibitors. The amount of mRNA transcripts specific of TNAP are shown in panel (C).

We next investigated whether the TNAP interacting with PrP^C^ at the cell surface of 1C11^5-HT^ and 1C11^NE^ cells was functional. To preserve at best the TNAP ectoenzyme natural microenvironment, we developped a chemiluminescence assay using the CSPD probe. It allowed us to measure under physiological conditions, TNAP and other phosphatase activities present at the cell surface of adherent live cells. As shown in Figure 6C (white bars), phosphatase activities monitored in 1C11 precursor cells were much lower (3 to 4 fold) than in fully differentiated 1C11^5-HT^ neuronal cells. We sought to specify whether the increase in phosphatase activity associated to neuronal differentiation could be attributed to TNAP. To this purpose, the chemiluminescent assay was performed in the presence of orthovanadate (1 mM), a phosphatase inhibitor with broad specificity, or tetramisol (5 mM), a specific inhibitor of the TNAP enzyme. Interestingly, TNAP has the particularity of being inhibited by tetramisol but not by orthovanadate [32]. While not affected by tetramisol (grey bar), exposure of 1C11 undifferentiated cells to orthovanadate (black bar) fully switched off the phosphatase activity indicating that a set of phosphatases, distinct from TNAP, is present at the neuroectodermal precursor stage. By contrast, in 1C11^5-HT^ serotonergic cells, tetramisol inhibited around 65% of phosphatase activities. The remaining activity corresponded roughly to the level already present in undifferentiated 1C11 cells (Fig. 6C). Noticeably, in 1C11^5-HT^ cells, around 65% of the phosphatase activity which is resistant to orthovanadate fully relates to TNAP. Similar phosphatase profiles were obtained with 1C11^NE^ cells (see Fig. 7B).

The time of onset of a functional TNAP among total phosphatase enzymatic activity was then monitored during the kinetics of serotonergic and noradrenergic differentiation of 1C11 cells (Fig. 7). This is rendered possible by the synchronicity and the homogeneity of differentiation of 1C11 cells. In 1C11^5-HT^ serotonergic and 1C11^NE^ noradrenergic differentiating cells, phosphatase activity levels kept increasing during the time course of both neuronal differentiation programs, till completion (day 4 for 1C11^5-HT^ and day 12 for 1C11^NE^ cells). Such an increase in cell surface phosphatase activities was majorly attributable to an induction of TNAP as demonstrated by sensitivity to tetramisol (Fig. 7A and B). This TNAP activity accounted for 60–70% of total phosphatase activities in differentiated cells. Of note, the induction of a TNAP enzymatic activity during 1C11 bioaminergic differentiation fully matches the kinetics of expression of TNAP specific mRNA (Fig. 7C).

These results demonstrate that a functional TNAP is induced as early as day 3 of both the serotonergic and noradrenergic neuronal pathways and reaches a maximal activity upon implementation of a complete bioaminergic phenotype. The onset of TNAP activity at the surface of bioaminergic cells, which precedes the implementation of a complete phenotype, may confer to this phosphatase a role in the modulation of neuron- or neurotransmitter-associated specialized functions.

### TNAP is involved in the control of serotonin and catecholamines synthesis

The specific role of TNAP in the CNS is still elusive. TNAP is known to function as an ectoenzyme to convert pyridoxal phosphate (PLP) into pyridoxal (PL), ensuring the passive uptake of this non-phosphorylated form of vitamin B6 into the cells where PL is converted back to PLP by intracellular kinases. In neuronal cells, PLP is an essential cofactor of the decarboxylases required for neurotransmitter synthesis i.e. glutamate decarboxylase (GAD) for GABA and amino acid decarboxylase (AADC) for bioamines. To date, an involvement of TNAP has been inferred in GABAergic neurotransmission only [33]. A potential link between a TNAP–dependent control of vitamin B6 metabolism and serotonin (5-HT) or catecholamine (CA) levels has not been established. We evaluated the impact of TNAP inhibition on 5-HT and CA synthesis in 1C11^5-HT^ and 1C11^NE^ cells. Cells having implemented a complete phenotype (day 4 for 1C11^5-HT^ and day 12 for 1C11^NE^) were exposed to tetramisol (2.5 mM) for up to 6 hours and cell extracts were collected at various time-points to measure the levels of bioamines and their precursors.

**Figure 8:**
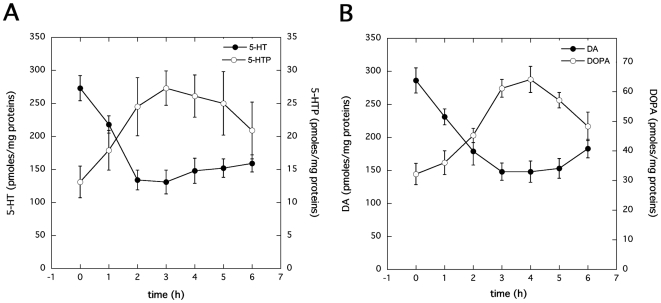
Involvement of TNAP in serotonin and catecholamine synthesis. (A) The intracellular content of 5-HT and its precursor 5-hydroxytryptophan (5-HTP) were measured in 1C11^5-HT^ cells treated with 2.5 mM tetramisol for up to 6 h. (B) 1C11^NE^ cells were exposed to 2.5 mM tetramisol for up to 6 h and their intracellular content of dopamine (DA) and its precursor dihydroxyphenylalanine (DOPA) were measured and expressed as pmoles/mg of total protein. Data represent the means ± S.E. of three independent experiments performed in triplicate.

As shown in Figure 8, tetramisol promoted a significant decrease in 5-HT (2 fold) or dopamine (DA) (1.8 fold), i.e. the AADC products, concomitant with an increase of their precursors 5-hydroxytryptophan (5-HTP) and dihydroxyphenylalanine (DOPA), respectively. This effect was observed as soon as 1 h, peaked after 2 h, remained stable over 6 h (Fig. 8A and B) and vanished after an overnight treatment (data not shown).

These data provide direct evidence that TNAP activity may act on 5-HT and CA synthesis in 1C11^5-HT^ and in 1C11^NE^ cells and define TNAP as a player in neurotransmitter metabolism.

### TNAP modulates the phosphorylation state of laminin and its binding to PrP^C^, in both 1C11^5-HT^ and 1C11^NE^ cells

**Figure 9:**
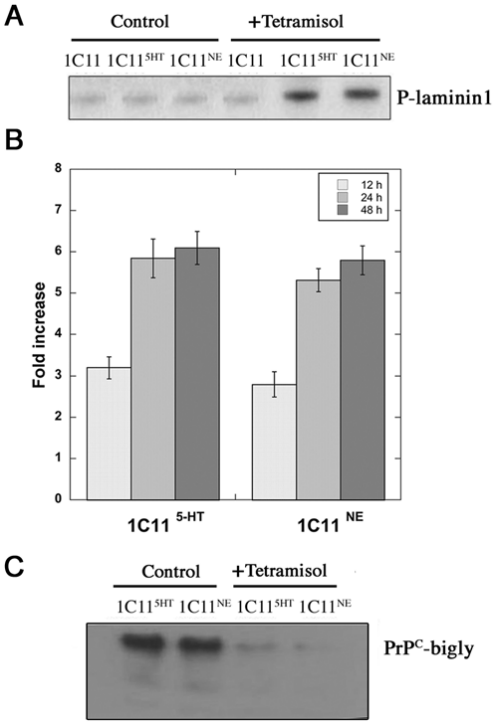
TNAP modulates laminin phosphorylation and binding to PrP^C^ in 1C11^5-HT^ and 1C11^NE^ differentiated cells. The phosphorylation level of Laminin 1, isolated from spent medium of undifferentiated 1C11 cells or its neuronal 1C11^5-HT^ serotonergic and 1C11^NE^ noradrenergic derivatives pre-incubated with [γ-^32^P]-ATP, was detected by autoradiography as shown in (A) for untreated cells (control) or at 48 h upon TNAP inhibition with tetramisol (+ tetramisol (2.5 mM)). In (B) the fold increase of phopho-laminin in tetramisol treated vs untreated cells was quantified at different times with a PhosphorImager and the values correspond to the mean of three independent experiments. (C) Laminin-1 was immunoprecipitated from 1C11^5-HT^ serotonergic and 1C11^NE^ noradrenergic cells left untreated (left) or treated for 24-hr with 2.5 mM tetramisol (right) and immunodetection was carried out with anti-PrP antibodies (SAF32).

While TNAP activity on phospho-monoesters is well established, there are only few reports suggesting that TNAP could act on phospho-proteins. TNAP might in fact exert opposite action to ecto-kinases on extracellular matrix (ECM) substrates. Based on this assumption, we probed the impact of TNAP inactivation on the phosphorylation of laminin, selected as a read out as both a target of ecto-kinases [34] and a PrP^C^-partner [16]. As shown in figure 9A, laminin was barely phosphorylated in 1C11, 1C11^5-HT^ and 1C11^NE^ control cells. As anticipated from the lack of TNAP expression in 1C11 precursor cells, the level of laminin phosphorylation was insensitive to tetramisol. In contrast, exposure of 1C11^5-HT^ and 1C11^NE^ bioaminergic neuronal cells to 2.5 mM tetramisol promoted a raise in laminin phosphorylation. A five fold increase in the amount of phospho-laminin was quantified at 24 h, that persisted over 48 h in 1C11^5H-T^ and 1C11^NE^ treated cells vs untreated cells (fig. 9B).

Immunoprecipitation experiments were further carried out to evaluate the possible impact of laminin phosphorylation on its interaction with PrP^C^. In agreement with the work of Graner [16], PrP^C^ was found to associate with laminin in 1C11^5-HT^ and 1C11^NE^ cells (fig. 9C, left panel). Upon exposure of 1C11^5-HT^ and 1C11^NE^ cells to 2.5 mM tetramisol for 24 h, the interaction between laminin and PrP was nearly lost (fig. 9C, right panel). As a whole, these results identify laminin as a target of the PrP^C^-interacting partner TNAP in neuronal cells. We may also conclude that, by modulating the phosphorylation level of laminin, TNAP impacts on the interaction between PrP^C^ and laminin.

## Discussion

In the present work, we identify the tissue non-specific alkaline phosphatase, TNAP, as a partner of PrP^C^ in lipid microdomains of 1C11-derived bioaminergic neuronal cells. This was established through co-immunoprecipitation and mass spectrometry analyses. Three major observations relate to this partnership: (i) the PrP^C^-TNAP interaction is restricted to the 1C11^5-HT^ and 1C11^NE^ neuronal progenies, (ii) it occurs in lipid rafts where both protagonists, which are GPI-anchored, preferentially reside, and, (iii) inhibition of TNAP activity alters the phosphorylation state of the PrP^C^-binding protein laminin, suggesting that PrP and TNAP could functionally interact.

The 1C11 neuronal differentiation model used in the present study has already allowed to gain information on PrP^C^ function. Besides a ubiquitous intracellular signaling coupled to PrP^C^ involved in red-ox equilibrium and cell homeostasis [22], our previous findings have uncovered some neuronal specific function of PrP^C^. This first relates to the selective implementation of a PrP^C^-caveolin-Fyn platform governing several signaling pathways converging on ERK1/2 in the differentiated 1C11^5-HT^ and 1C11^NE^ neuronal cells [3], [22]. A second neurospecific role of PrP^C^ is to modulate serotonin receptor intracellular coupling and crosstalks [25]. Remarkably, both the proper instruction of signal transduction events by PrP^C^ and its interference with serotonin receptor responses involve caveolin. These observations illustrate the functional implication of PrP^C^ location in a subtype of lipid rafts, the caveolae, involved in cell signaling and capable of internalizing membrane receptors. However, the cellular and molecular basis accounting for PrP^C^ neurospecific function still has to be characterized. It could rely on the recruitment of a selective subset of PrP^C^ isoforms in lipid rafts. An alternative explanation would be the involvement of additional molecules whose expression and/or interaction with PrP^C^ is restricted to mature neuronal cells. In this context, the present identification of TNAP as a neurospecific PrP^C^ partner posits TNAP as one such a candidate.

Besides, our results support the notion that the onset of a functional TNAP accompanies the serotonergic and noradrenergic differentiation of 1C11 cells. This is substantiated by (i) the expression of TNAP mRNAs in the differentiated progenies of the 1C11 cell line and the lack of transcripts in 1C11 precursor cells, (ii) the selective implementation of a tetramisol-sensitive TNAP activity during the kinetics of differentiation coinciding with TNAP protein expression and, (iii) the participation of this ectophosphatase to neurotransmitter metabolism. This latter observation is in line with the well-established TNAP-mediated regulation of pyridoxal phosphate (PLP), a cofactor of decarboxylases contributing to the last step of some neurotransmitter synthesis (serotonin, norepinephrine, GABA…). This TNAP associated phosphomonoesterase activity may confer an important role to this protein in the nervous system, as discussed below.

Noteworthy, our experimental design based on lipid raft isolation shows that PrP^C^ and TNAP interact within these specialized microdomains in which they segregate. The location of PrP^C^ in lipid rafts or its interaction with molecules in such microdomains has been described using other approaches. For instance, Schmitt-Ulms et al. have investigated into PrP^C^ partners in total brain samples. Their analysis confirms that PrP^C^ resides in a membrane environment containing proteins specific of lipid rafts and, in particular, a subset of molecules that, like PrP^C^, use a GPI-anchor [35]. PrP^C^ interacts with GM3 gangliosides present in high amount in lymphocyte and neuronal lipid rafts [36], [37] and with other glycoproteins or glycolipids [38]–[40], which co-localize or are enriched with PrP^C^ in rafts of neuronal cells. Whether these partners participate in PrP^C^ function is however unknown. Noticeably, different intracellular signaling molecules such as kinases and adaptors, recruited through lipid rafts, have been implicated in PrP^C^ functional interactions [3], [13], [20], [37], [41], [42]. Although the functional relevance of PrP^C^ compartimentation within rafts has been poorly addressed, it has recently been established that PrP^C^ does recruit NCAM into lipid rafts where it instructs Fyn activation and subsequent neurite outgrowth and neuronal polarization [20]. Our present identification of TNAP as a novel raft-specific PrP^C^ interacting molecule adds further weight to the idea that PrP^C^ location in rafts deals with its neuronal function. It is now well established that lipid rafts constitute dynamic sub-membrane structures allowing the concentration of specific lipids, glycolipids and glycoproteins serving particular functions [43]. In view of the increasing set of molecules described as interacting with PrP^C^ in membrane microdomains, it is tempting to speculate that PrP^C^ takes part to multi-molecular complexes whose onset is favored by the specific lipid local composition and which may sustain signal transduction events. Further investigation will be required to determine whether TNAP functional interaction with PrP^C^ occurs directly or indirectly via the intermediate of other proteins related to neuronal differentiation programs.

**Figure 10:**
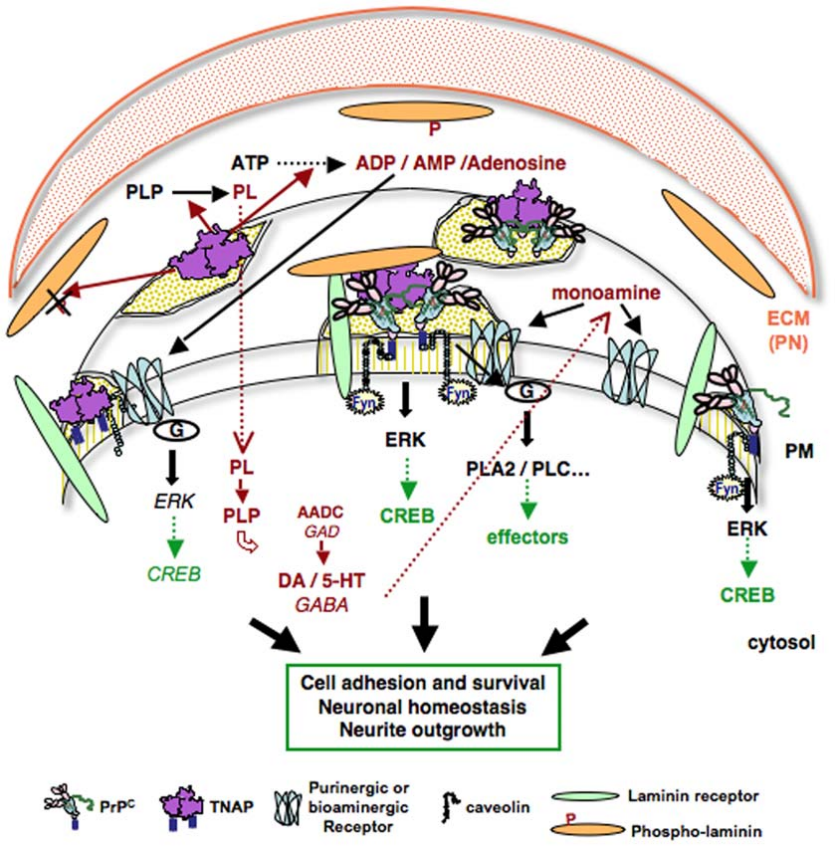
Diagram depicting possible implications of a PrP^C^-TNAP association in membrane microdomains of neuronal cells. PrP^C^ and TNAP are GPI-anchored membrane proteins, which majorly reside in rafts. Both have been described to interact with ECM proteins [16], [53], [54] and to participate to cell signaling events. PrP^C^ can instruct downstream signaling events, including ERK and CREB activation, by mobilizing a Cav/Fyn complex on neurites [3], [22]–[24]. In addition, it modulates the coupling of 5-HT receptors, with specific impact according to G protein-dependent pathway [25]. The TNAP ectophosphatase may have different substrates. (i) By promoting PLP hydrolysis it contributes to the regulation of neurotransmitter synthesis [33]. (ii) Its nucleotidase activity may have implications for purinergic signaling [30], [46]–[48]. (iii) TNAP may be active on phosphoproteins notably of the cell surface [51], [52]. The identification of phospho-laminin as a TNAP substrate uncovers a novel role of this ectoenzyme in the regulation of ECM molecules. Laminin and the laminin receptor are important components of the perineural net (PN) and are known partners of PrP^C^. The interplay between PrPC, laminin and TNAP within multiprotein complexes may have implications for neuronal functions (survival, homeostasis, plasticity).

An interaction of PrP^C^ with TNAP may have different implications in neuronal cells in relation to the various roles envisioned for this ectoenzyme (see fig. 10). TNAP is a homodimeric metalloenzyme that hydrolyses phospho-monoester specific substrates, phosphoethanolamine (PEA), inorganic phosphate (PPi), an important player in bone mineralization, and pyridoxal phosphate (PLP), a cofactor of decarboxylases contributing to neurotransmitter synthesis. However, little is known about the role of TNAP under physiological conditions and it is only recently that this ecto-phosphatase has been recognized to be important in the nervous system [29], [44]. A role of TNAP in neurotransmission is well illustrated by the observation that TNAP knock-out mice develop epilepsy due to GABA deficiency [33]. These defects recall the occurrence of seizures in patients with mutations in the *ALPL* gene, suffering from severe hypophosphatasia. Moreover, recent data show that TNAP activity is regulated by sensory experience [29]. Since serotonin containing fibers are present at high density in sensory regions of the brain, the authors suggest that TNAP could also regulate serotonin or dopamine synthesis and participate in cortical function and neuronal plasticity by regulating neurotransmitter synthesis. Our data indeed establish a link between TNAP activity and bioamine synthesis in 1C11^5-HT^ and 1C11^NE^ cells. Hence the interaction of PrP^C^ with TNAP may confer to the prion protein a role in neurotransmitter homeostasis and neuronal transmission. In this regard, it is worthy to note that TSE-associated neurodegeneration is accompanied by alterations in neuronal transmission notably involving the serotonergic system [45].

Besides, TNAP could contribute to ectonucleotidase activity in the brain [30], [46], [47]. Indeed, TNAP has the capacity to dephosphorylate ATP to adenosine in a stepwise manner [48]. Nucleotide signaling exerts important neuronal function in the development of the nervous system and in synaptic transmission in adult brain [44]. Interestingly, a change in nucleotidase activity has been detected in PrP^C−/−^ mice which exhibit a slower rate of ADP hydrolysis possibly leading to a lower level of adenosine [49]. Adenosine has an anticonvulsant effect and this has to be put together with the recent observation that such PrP^C^ deficient mice are more prone to develop seizures in response to convulsant compounds [50]. The susceptibility to seizures and epilepsy recalls the phenotype of TNAP knockout mice. Possibly, defects in TNAP activity could account for some of the changes in brain ectonucleotidase activities reported in hippocampal and cortical synaptosomes of mice lacking PrP^C^ [49]. Further investigation into TNAP activity in a PrP^C^ null context should help clarify this issue.

Beyond its phospho monoesterase and ectonucleotidase activity, TNAP may also exert a phosphatase activity on proteins [51]. This is notably supported by the demonstration by Becq et al that TNAP inhibition enhances the phosphorylation and concomitant activation of the Cystic Fibrosis Trans-membrane receptor (CFTR) [52]. Interestingly, this ectoenzyme could also have a role on extracellular matrix proteins, as supported by its collagen-binding domain [53], [54]. In line with this, our data define phospho-laminin as a TNAP substrate in both 1C11^5-HT^ and 1C11^NE^ neuronal cells. To our knowledge, this is the prime evidence that TNAP may contribute to regulate the phosphorylation state of an ECM protein in neuronal cells. In contrast, the partnership between PrP^C^ and laminin has raised much attention over the past few years. The interaction of PrP^C^ with laminin has been shown to sustain both neurite outgrowth [16], neuronal differentiation of PC12 cells [55] and memory consolidation [56]. Whether these processes are modulated according to the phosphorylation state of laminin remain to be investigated. Our data support the notion that the phosphorylation level of laminin influences its ability to interact with PrP^C^ and define TNAP as a novel protagonist in the PrP^C^-laminin interplay. They add to the current notion that PrP^C^ may be part of large multi-molecular complexes, depending on the cellular context and environment, and thereby contribute to diverse cellular functions [57]. Resolving the complexity of PrP^C^ partners and functional interactions in neuronal cells should lead to a better understanding of the neurospecificity of PrP^C^ function.

## Materials and Methods

### Cell culture and reagents

1C11 cells were grown in DMEM medium (Gibco) supplemented with 10% foetal calf serum (Seromed) and, differentiation into serotonergic (1C11^5-HT^) or noradrenergic (1C11^NE^) neuronal cells was induced respectively by addition of dibutyril cyclic AMP (dbcAMP) or addition of dbcAMP in presence of 2% DMSO as previously described [21]. Unless stated otherwise, 1C11^5-HT^ cells correspond to day 4 of serotonergic differentiation and 1C11^NE^ cells correspond to day 12 of noradrenergic differentiation. The BW5147 mouse myeloma cell line was grown in RPMI containing 7.5% foetal calf serum (Gibco). For inhibition of TNAP, tetramisol was added as indicated in the culture medium. Unless indicated, the reagents were purchased from Sigma.

### Antibodies

Mouse monoclonal antibodies specific of prion protein were from SPI-BIO. SAF32 and SAF34 antibodies recognize an N-ter epitope (a.a. 79–92) while Bar221 is specific of the C-ter region of PrP^C^ (a.a. 140–160). N-CAM was revealed using an anti-pan N-CAM mouse monoclonal antibody (BD Bioscience). We also used mouse monoclonal anti-caveolin and anti-flotillin antibodies (Transduction Laboratory) and a rabbit polyclonal antibody to Lck (Upstate). Preparation of the anti-TNAP antibody as been previously described [31]. Antibody MAB2549 against Laminin-1 was from R&D systems.

The secondary reagents used for immunoblot detection were, either goat anti-mouse or goat anti-rabbit antibodies coupled to horseradish peroxidase (HRP) accordingly to the primary antibody, or streptavidin HRP to detect biotinylated proteins in immune complexes and were all purchased from Southern Biotechnology. The secondary antibodies (Molecular Probe) used in immunofluorescence were a goat anti-mouse and a goat anti-rabbit antibodies coupled to alexa fluo 488 (green) and alexa fluo 594 (red), respectively.

### Preparation of lipid rafts (GSL) on sucrose gradient and cell surface biotinylation

Purification of the glycosphingolipid (GSL) rich complexes was performed as described for lymphoblastoid cells [58]. 1C11 adherent cells were washed twice in PBS then scraped on ice in a small volume of PBS containing a cocktail of protease inhibitors (Complete™ from Roche) and 1 mM sodium orthovanadate (Na_3_VO_4_) phosphatase inhibitor. Around 10^8^ cells were disrupted and homogenized at 4°C in 3 ml of MBS (Mes buffered saline): 25 mM Mes pH 6.5; 150 mM NaCl, containing 1% triton X-100 (Tx100), phosphatase and protease inhibitors. The homogenate (HT) was clarified by 1 min centrifugation at 1000 rpm and brought to a volume of 4 ml at 40% sucrose in MBS-Tx100. The homogenate in 40% sucrose was transferred to a Sw41 tube (Beckman), overlaid with 4.5 ml of a 30% sucrose solution in MBS (without triton) then, with 2.7 ml of a third layer containing MBS without sucrose. The step-gradient was centrifuged for 20 h at 180000 g and at 4°C in a Sw41 rotor (Beckman). The lipid rafts containing GSL complexes appear as an opaque band 5 mm beneath the 0%–30% layers interface. They were harvested and diluted to a volume of 3 ml in MBS. GSL complexes were then pelleted by centrifugation for 1 h at 300000 g in a TL100.3 rotor (Beckman). Such raft preparations were dissolved in 6% SDS-RIPA buffer (150 mM NaCl, 25 mM Tris HCl pH 7.4, 5 mM EDTA, 0.5% Na-DOC and 0.5% NP40). The different soluble fractions at 40% (F40) and 30% (F30) sucrose as well as the triton insoluble high-speed pellet (HSP) were collected from the gradient and their protein concentration was determined using BCA kit (Pierce).

For analysis of PrP^C^ partners in lipid rafts, membrane proteins were biotinylated prior to GSL preparation. Cells in monolayer were washed twice with PBS Ca^2+^/Mg^2+^ then incubated with EZ-link™-sulfo NHS-LC-biotin (Pierce) at a concentration of 0.5 mg/ml in PBS for 30 min at 4°C to limit endocytosis of membrane receptors. Adherent biotinylated cells were washed and GSL were isolated as above, except they were diluted in NET buffer (150 mM NaCl, 50 mM Tris HCl pH 7.4, 5 mM EDTA) containing 1% Tx-100 (Calbiochem) and heated 1 h at 37°C in order to improve solubilisation of proteins embedded into cholesterol and to allow further immunoprecipitation of PrP^C^ complexes.

### Immunoprecipitation and western blot analysis

Specific immunoprecipitations were performed using protein A or protein G sepharose beads covalently linked to anti-PrP^C^ IgG2a (SAF34) or IgG1 (Bar221) respectively. This procedure avoids recovering of IgG in the complexes which is of importance for MS analysis. We used the Seize™-X protein A (or G) immunoprecipitating kit (Pierce) to prepare immunoabsorbant according to the manfacturer's recommandations. Anti-PrP coupled-beads were then incubated overnight at 4°C with biotinylated rafts in lysis buffer containing Tx-100. Beads were washed 4 times in high salt buffer (NET, 1% Tx100 in 0.5 M NaCl), then twice in Hepes 40 mM before elution of the immune-complexes in a reducing sample buffer containing SDS. For analyses in western blot, 2.5 µg of raft proteins were immunoprecipitated while for further purification of PrP^C^ partners for mass spectrometric analysis, a high amount of raft was used (equivalent to 20–30 µg). Denatured complexes were run on SDS-PAGE (Bio-Rad). After transfer of proteins from the gel onto nitrocellulose membrane (Amersham), the membrane was blocked with 1% gelatin in PBS 0.1% Tween 20 (PBST). Detection of PrP^C^ and associated proteins was performed using streptavidin-HRP (Southern Biotechnology) 1/100 000 and the ECL chemiluminescent procedure (Amersham).

The same SDS-PAGE and western blot procedures were used to directly detect proteins in 15 µg of total extract prepared in NET-Tx100 lysis buffer or in 1 µg of raft proteins prepared in 6% RIPA buffer. After blocking, membranes were reacted with the specific primary antibodies *i.e.* SAF32 (10 µg/ml), anti-N-CAM (2 µg/ml), anti-caveolin (0.05 µg/ml), anti-flotillin (1 µg/ml), anti-Lck (0.1 µg/ml), anti-TNAP (1/400). Immunoblots were revealed by specific secondary antibodies coupled to HRP (1/10000) before ECL staining.

To probe an interaction of PrP^C^ with laminin, 1C11^5-HT^ and 1C11^NE^ cells were incubated with antibodies against laminin-1 (10 µg/ml) in PBS containing 0.5% BSA for 1 h at 4°C. Cells were washed twice with PBS Ca^2+^/Mg^2+^, scrapped and collected by centrifugation (10,000 g, 3 min, 4°C). Pellets were resuspended in NET lysis buffer containing 1% Tx-100. Lysates were transferred onto protein-A sepharose beads and the last steps of immunoprecipitation were carried out as described above. SAF32 antibodies were used to detect PrP.

### Mass spectrometry

Peptides were generated for mass spectrometry analysis by in-gel trypsin digestion of proteins. Since the gel was not stained, we used pre-stained standard molecular weight as reference to evaluate the 80 kDa position. One mm large gel slices excised from 8% SDS-PAGE and including proteins of interest with an apparent molecular mass of 80 kDa, were reduced with DTT and alkylated by iodoacetamide treatment. The enzyme digestion was carried out overnight at 37°C with modified sequencing grade trypsin (Promega, Madison, WI). Peptides were then extracted from the gel by treatment with a solvent solution containing 5% formic acid and 50% acetonitrile. The extracts were dried under vacuum and re-suspended in a minimum volume (10 µl) of a solution at 0.1% formic acid and 5% acetonitrile and 4 µl of peptide extract were analysed.

Mass spectrometric analyses were performed by LC-ESI-MS/MS where a nanoflow liquid chromatography (LC-Packings nanoflow LC system, Dionex Inc) is coupled to a nano electrospray ionisation system (ESI) and a tandem mass spectrometer (MS/MS) analyser (Deca XP LCQ-Ion trap mass spectrometer instrument, Thermo Electron, Waltham, MA). The system allows peptide extracts to be desalted and concentrated on a capillary peptide trap (1 mm×300 µm ID) prior to injection on a C18-resin (LC-Packings, Netherlands) column (15 cm×75 µm ID pepMap column). Peptides were eluted at a constant flow rate of 170 nl/min by applying a discontinuous acetonitril gradient (5%–95%). The column exit is directly connected to the nanoelectrospray ion sources and the instrument is operated in data-dependent acquisition mode to automatically switch from MS to MS/MS analysis. MS/MS spectra were obtained by fragmenting ion peptides by collision-induced dissociation (CID) using normalized collision energy of 30% in the ion trap.

The data files generated by LC-MS/MS were converted to Sequest generic format files and were confronted to the *mus musculus* NCBI non-redundant database using Bioworks 3.1 Search Engine (ThermoFinnigan). Search parameters for determination of peptide sequences included carbamidomethyl as fixed modification and oxidized methionine as variable modification.

### PCR analysis

cDNA were reverse-transcribed using the superscript ™II RT kit (Invitrogen) from 5 µg of mRNA prepared from 1C11 cells and its neuronal derivatives by Mini-Prep column purification (Quiagen). TNAP PCR specific fragment was obtained by 23 cycles of amplification at 62°C in a thermocycler PTL-100 (MJ research) using the following specific primers: sense = 5′-GCAGGATTGACCACGGACACTATG-3′; anti-sense = 5′-TTCTGCTCATGGACGCCGTGAAGC-3′. As an internal control, GAPDH was amplified by 18 cycles at 58°C with the 2 primers: sense = 5′-TGAAGGTCGGTGTGAACGGATTTGGC-3′; anti-sense = 5′-CATGTAGGCCATGAGGTCCACCAC-3′. Specific GAPDH and TNAP fragments were run on 1% and 1.5% agarose gel respectively and revealed by BET staining.

### Enzymatic activity of alkaline phosphatase

Phosphatase activity was determined at the surface of intact cells performing enzymatic test on cells that were cultured in 96 wells-microplates. Cell layers were washed twice with PBS Ca^2+^/Mg^2+^ then incubated with CSPD chemiluminescent substrate (Roche) at a concentration of 0.25 mM in 200 µl of a physiologic buffer (135 mM NaCl, 4 mM KCl, 1 mM CaCl_2_, 20 mM Hepes pH 7.5, 5 mM glucose and 1 mM MgCl_2_) as described [59]. In order to discriminate between different phosphatase activities, the substrate was reacted with cells with or without 5 mM tetramisol, which inhibits TNAP and with or without 1 mM Na_3_VO_4_ which exhibits a larger spectra of phosphatase inhibition, but is not active on TNAP. Each condition was tested in 6 replicates. Chemiluminescence amplification resulting from phosphohydrolysis of the CSPD substrate was monitored in a Perkin Elmer reader plate. The data are given as relative luminescent units (RLU/µg prot/h).

### Membrane immunofluorescence

1C11 cells were cultured on glass cover slips at the bottom of 24 wells-micro plates and induced to differentiate into 1C11^5-HT^ cells. Membrane immunofluorescence was carried out on intact cells reacted for 1 h at room temperature with SAF32 anti-PrP (10 µg/ml) and anti TNAP (1/100) antibodies diluted in PBS Ca^2+^/Mg^2+^, 2% FCS and 0.1% sodium azide to avoid internalization of membrane receptors. After 3 washes in PBS/azide, secondary fluorescent antibodies were added for 1 h. After washing, cells were fixed with 3.7% formaldehyde then mounted in fluoromount (Southern Biotechnology). Examination was carried out on an Axiophot microscope (Zeiss) equiped with UV lamp and appropriate emission filters for epifluorescence and with a camera (Nikon) and video system (Packard Bell). In addition, sequential acquisition was performed on a scanning confocal microscope (Leica confocal SP5) at 405, 488 and 561 nm.

### Determination of cellular content of bioamines and bioaminergic precursors

1C11^5-HT^ or 1C11^NE^ cells grown in DMEM supplemented with 10% 5-HT-depleted FCS were exposed to 2.5 mM tetramisol for up to 24 hours. This tetramisol concentration allows to fully abrogate TNAP activity (Fig. 7) and lacks any cell toxicity (data not shown). Cells were washed twice with PBS, scrapped and collected by centrifugation (10,000 g, 3 min, 4°C). The levels of serotonin (5-HT), dopamine (DA) and their precursors 5-hydroxytryptophan (5-HTP) and dihydroxyphenylalanine (DOPA), respectively, were measured by HPLC with a coulometric electrode array (ESA Coultronics, ESA Laboratories, Chelsford, MA), as in [60]. Quantifications were made by reference to calibration curves obtained with internal standards.

### Phosphorylation of laminin

The phosphorylation state of endogenous laminin was assessed by measuring [γ-^32^P]-ATP incorporation (specific activity 18.5 Gbq/mmol, Amersham Pharmacia Biotech). Briefly, 1C11, 1C11^5-HT^ or 1C11^NE^ cells were grown in roller bottles in serum free conditions. [γ-^32^P]-ATP (1.2 GBq per 10^6^ cells) was added to the culture medium 1 hour prior tetramisol (2.5 mM) addition. Spent medium was collected at various time points following tetramisol treatment, concentrated by ammonium sulfate at 80% saturation and dialyzed against 20 mM Tris-HCl pH 7.5, 0.5 M NaCl, 0.005% Brij-35 (TNB buffer). Laminin 1 was purified from the concentrated conditioned medium through affinity chromatography using a protein A-Sepharose column (Biorad) chemically conjugated with anti-laminin 1 antibody. Following elution, samples were run on a 7% SDS-PAGE and incorporation of radiolabeled phosphate was quantified using a PhophorImager (Molecular Dynamics).
